# Neural indicators of sleep loss and sleep propensity in male military trainees: Insights from dry‐electrode EEG—An exploratory study

**DOI:** 10.14814/phy2.70301

**Published:** 2025-03-28

**Authors:** Jazmin Morrone, Sarah Coakley, Sam Blacker, Stephen Myers, Paul Hough, Christopher Vine, Tessa Maroni, Neil Stanley, Shona Halson, Andrew Siddall, Stephen Patterson, Martin Jones, Kieran Chillingsworth, Charles R. Pedlar

**Affiliations:** ^1^ Faculty of Sport, Allied Health, and Performance Science St Mary's University, Twickenham London UK; ^2^ Institute of Applied Sciences University of Chichester Chichester UK; ^3^ Oxford Brookes University Oxford UK; ^4^ Independent Sleep Expert Farnborough UK; ^5^ Australian Catholic University Brisbane Australia; ^6^ Defence Science and Technology Laboratory (Dstl) Salisbury UK

**Keywords:** dry‐electrode, fatigue, oculography, odds ratio product, psychomotor vigilance task

## Abstract

This study examined the impact of reduced sleep on electroencephalogram (EEG) activity during cognitive tasks in Military Clearance Diver trainees using a novel dry‐electrode EEG system. Seven male participants underwent two 5‐day periods: a baseline and a “live‐in” phase with increased workload and reduced sleep (5.4 ± 0.1 vs. 7.4 ± 0.7 h). EEG was recorded daily in the early morning (am) and late afternoon (pm) during a Psychomotor Vigilance Test (PVT), two oculography tests (am: *n* = 4; pm: *n* = 3), and 2 min of eyes‐closed rest. Significant increases in theta (*t* (29) = 2.308, *p* = 0.028, *d* = 0.421) and alpha (*t* (29) = 2.124, *p* = 0.042, *d* = 0.388) power spectrum densities were observed in the “live‐in” phase during the PVT. These findings align with increased lower frequency activity over time awake, reflecting heightened sleep propensity. Sleep loss was further confirmed by declining Odds Ratio Product (ORP) values. This study demonstrates the feasibility of dry‐electrode EEG in detecting fatigue‐related neural changes and highlights the potential of ORP as a quantifiable fatigue marker. These insights may inform operational settings, such as military diver performance monitoring and fatigue management strategies.

## Graphical abstract

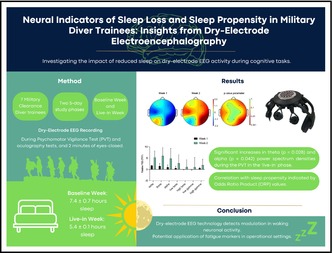

## 1 INTRODUCTION

Within military populations, high degrees of sustained attention and alertness are essential for the execution of daily operational tasks (Nindl et al., 2018). This is a particularly important requirement during hostile or high‐stress environments, as these often involve lethal parameters and serious ramifications. Within the defense sector, both training and operation days often involve high degrees of heavy physical and cognitive workloads paired with long working hours (Nindl et al., 2018). Successfully detecting and monitoring performance deterioration associated with dangerous states of fatigue (i.e., due to sleep deprivation [SD]) can help to prevent the costly or even life‐threatening consequences associated with human performance decline (Lieberman et al., 2006; Williams et al., 2014).

The establishment of both subjective (i.e., scales of sleepiness/fatigue such as the Karolinska Sleepiness Scale [KSS] (Åkerstedt & Gillberg, 1990)) and objective (e.g., Psychomotor Vigilance Task [PVT]) markers have been widely studied and validated as tools that can be used to identify states of inadequate sleep (Lim & Dinges, 2008; Shen et al., 2006). Such markers of SD have been found to correlate with deficits in simple cognitive task performance, including attention and alertness, reaction time, and vigilance (Dinges & Kribbs, 1991; Shen et al., 2006), and complex cognitive task performance (i.e., executive functions and decision making (Jones & Harrison, 2001)). As the temporal dynamics associated with vigilant attention (i.e., which may be quantified with the PVT) are regulated by the dynamics involved in both homeostatic and circadian processes (Doran et al., 2001), one of the most popularized and most widely validated measures of SD is the PVT (Lim & Dinges, 2008).

The PVT is a computerized task that requires continuous attention to respond to stimuli occurring at random, which importantly neglects the opportunity of aptitude and learning (Durmer & Dinges, 2005). The PVT measures changes in vigilance, that is, sustained alertness, attention, and reaction time (Lim & Dinges, 2008), and has a proportionally high sensitivity to variations in acquired sleep corresponding to states of fatigue, sleep pathology, and adverse circadian phase acclimation/functioning (Durmer & Dinges, 2005). In particular, impaired performance, or slower and more variable PVT responses, have been repeatedly shown to correspond with sleep loss (Chua et al., 2014). Due to the validity associated with the PVT as a measure for SD, this indicates that the neural mechanisms associated with attention are readily detectable as a marker for SD (Durmer & Dinges, 2005; Ramakrishnan et al., 2016).

Another form of quantifying vigilance includes the monitoring of eye movements in the form of “ocularmetrics” (Anderson et al., 2013). This is due to how ocular activity is directly linked to functions of the central nervous system which correspond to vigilance and attention, such as to midbrain reticular formation activity (Kinomura et al., 1996). Therefore, eye tracking systems have become prominent in characterizing and defining states of SD due to the known correlation between vigilance and slower eye movements (Anderson et al., 2013). Although the relationship between SD with cognitive tests (such as PVT performance (Lim & Dinges, 2008)) have been established, less is known about the neural basis associated with cognitive performance as a result of reduced sleep duration, and thus, in sleep‐deprived states.

Electroencephalogram (EEG) studies have found nearly all frequency band spectral values to increase in states of SD compared to prior conditions, apart from the high‐beta frequency band (16–24 Hz) (de Gennaro et al., 2007; Finelli et al., 2000; Gorgoni et al., 2014; Tinguely et al., 2006). Although both the theta (4‐7 Hz) and alpha (8‐12 Hz) bands have been found to increase throughout the course of a wakefulness (Cajochen et al., 1995) and in states of SD (Corsi‐Cabrera et al., 1992; Horne & Baulk, 2004; Lorenzo et al., 1995; Smith et al., 2002), a particular emphasis on lower frequency bands, such as the delta band (0.5‐4 Hz) and the theta band (4‐7 Hz), have been made (Cajochen et al., 1999; Horne, 1993). This emphasis is due to these bands being most consistently found to increase with time elapsed since waking in parallel with sleep propensity (de Gennaro et al., 2007; Finelli et al., 2000; Gorgoni et al., 2014; Tinguely et al., 2006), and they have even been hypothesized to be a prominent feature of SD EEG (particularly within frontal regions (Cajochen et al., 1999; Horne, 1993; Oken et al., 2006)). Furthermore, the progressive increase in absolute and relative powers of lower frequency bands have been correlated with subjective sleepiness, slower eye movements, and reaction time (Lorenzo et al., 1995; Oken et al., 2006). More particularly, in states of SD, theta band powers have been found to increase during quiet/waking‐rest states and directly correlate to the degree of subjective sleepiness (Finelli et al., 2000; Smith et al., 2002; Strijkstra et al., 2003). Interestingly, the theta band has also been used to predict the subsequent increase in homeostatic sleep slow wave activity (SWA) (Vyazovskiy & Tobler, 2005).

While the relationship between EEG metrics and sleep stages is well‐established, less has been defined on the EEG‐based indices for arousal and restfulness during waking states. To address this, linearly defined and transferable metrics of arousal states hold promise for investigating the neural correlates of wakefulness. One such metric is the Odds Ratio Product (ORP), calculated within datasets as a continuous measure of wakefulness (Younes et al., 2015). The ORP quantifies the spectrum from very deep sleep (0) to full wakefulness (>2.5), offering an objective alternative to traditional sleep staging by removing subjectivity and capturing brief sleep episodes. By leveraging EEG frequency band powers, the ORP generates a single, reliable index of sleep and wake states through spectral analysis of EEG data. Therefore, integrating novel dry‐electrode EEG technology with a robust sleep–wake index, like the ORP, presents significant potential for detecting fatigue during wakefulness, particularly in operational settings such as the military. As the ORP has yet to be applied to waking EEG, the present study offers novel and valuable insight into the transferability of the tool for diversified applications.

Overall, this study aimed to provide insight on the neurological correspondences involved in wake‐dependent cognitive performance associating with states of reduced sleep duration, and employed a novel dry‐electrode EEG system as the selected neuroimaging technique for its feasibility and practical implementation into real‐world settings. Within SD literature, no studies to our knowledge have investigated the administration of both PVT and an eye‐tracking task (EyeSync) concomitantly with EEG‐based neuronal assessment. As such, the primary investigations of the study included: (1) investigating the neural correspondences associated with decreased PVT and EyeSync performance as a result of being in a state of reduced sleep duration, (2) evaluating the quantifiability of lower frequency bands as a measure for waking sleep deprived states, (3) investigating electrode site contribution of theta band spectral topographical distribution in waking states of increased restlessness, (4) quantifying changes in waking ORP values with increased workload and cumulative sleep loss. Based upon previous research, the following was hypothesized: (1) an increase in subjective propensity to sleep and decreased objective sleep duration will associate with increased overall waking‐rest theta activity, (2) within both cognitive tasks (PVT and eye‐tracking), lower frequency waves (i.e., delta and theta waves) will be most prominently impacted by the protocol compared to higher frequency waves, (3) theta activity will be higher in the pm compared to am in the “live‐in” week, due to the linear relationship the theta band has been found to have with time elapsed since waking (de Gennaro et al., 2007; Finelli et al., 2000; Gorgoni et al., 2014; Tinguely et al., 2006), (4) a decline in ORP values would be found during the test battery with cumulative sleep loss.

## 2 METHODS

### 2.1 Participants

This study investigated the EEG data acquisitions of seven male Military Clearance Diver trainees (M_age_ = 29.0 ± 3.0 years, M_height_ = 1.82 ± 0.06 m, and M_mass_ = 81.8 ± 4.8 kg) and was granted favorable opinion by the Ministry of Defence Research Ethics Committee (MoDREC, Protocol Number 2088/MODREC/21). Due to the specialist population that was eligible for recruitment, the sample size was limited by the number of participants available (total of eight personnel) to volunteer in the study. No power calculation was conducted since this was the entire intake of the specialized course. Potential participants were provided with written and verbal briefs of the protocol and procedure before being asked if they were willing to volunteer, wherein no uniformed personnel from the chain of commands were present. The information provided outlined any possible risks of the study, the right to withdraw from the study at any time and how the results would be used. Volunteers provided written informed consent prior to participating.

### 2.2 Study design

The study was a repeated measure design which consisted of two 5‐day periods of data collection with each day consisting of two scheduled discrete task measurements: an am and a pm session. The two consecutive week periods occurred in the latter weeks of the Military Clearance Diver course, which were broken into (1) a baseline phase and (2) a “live‐in” phase. The baseline phase involved the Military Clearance Diver trainees following normal working hours (08:00–16:00 h) which included: physical training, diving serials and classroom sessions, with the participants sleeping in their own homes. This baseline week was used to define normal variation in all variables for comparison with the “live‐in” (i.e., fatigued) phase. The “live‐in” phase of the Military Clearance Diver course is intended to simulate aspects of military operation, and as such, comprised of five consecutive days of high intensity operations and/or standby days and nights, therefore accumulating greater fatigue due to increase workload and decreased opportunity to sleep. For the purpose of this manuscript, the term *fatigue* is used to represent the combination of reduced sleep opportunity and increased physical workload. Both am and pm test sessions were undertaken in a controlled and sensory‐limited booths within a classroom with regulated temperature (20.7 ± 2.2°C) and light (332.5 ± 14 Lumens).

### 2.3 Procedure

The am sessions took place from 7:40 to 9:00 and the pm sessions from 15:40 to 17:00, with participants attending in the same order for each session for approximately 15 min each. Prior to each am session, sleep data were collected, and average sleep durations per night were calculated per participant. The am and pm sessions involved a discrete task protocol consisting of a 1‐min eyes open resting procedure, a 10‐min Psychomotor Vigilance Test (PVT) performed on a laptop and gaming mouse (Logitech G203, Logitech, Newark, USA), two oculography tests using the EyeSync device, and a 2‐min eyes‐closed procedure, respectively. This protocol was performed consecutively for time efficiency and simultaneously with continuous EEG monitoring. All cognitive tests were performed in both am and pm sessions, and EEG data were collected once per day per participant (i.e., consistently either the am or pm session). Four of the participants had their EEG data collected during the am session, and three during the pm session.

Once ready to commence, the selected participants were seated while the dry‐electrode EEG system was firmly placed and adjusted to fit each individual's scalp. Electrode impedance was monitored to ensure all electrodes detected a measurable signal. Once all electrodes showed sufficient impedance (<20kΩ.), EEG recordings commenced, and participants completed the discrete task protocol, as described above. In order to account for edge artifacts, buffer times consisting of a minimum of 3 s were implemented before and after each task. The 1‐min eyes‐open resting procedure was performed as a reference to normalize EEG data collections for each of the participants.

### 2.4 Materials

The data collections involved the use of a PVT (BHSAI, Frederick, MD (Reifman et al., 2018)), two oculography tests using an EyeSync device (SyncThink, Palo Alto, CA), and a dry‐electrode wireless 7‐sensor DSI‐7 EEG system (DSI‐7, Wearable Sensing, LLC, San Diego CA). The dry‐electrode EEG system has been used and shown to offer a reliable EEG signal (e.g., against gold standard EEG systems (Kohli & Casson, 2015; Mahdid et al., 2020; Snider et al., 2022)). The two oculography tests were a Smooth Pursuit test, which measured the participants eye motions (i.e., radial and spatial variance) and synchronization (i.e., timing and tangential variance) to a target; and a Horizontal Saccades Test, which focused on horizontal variance. EyeSync data were automatically processed using proprietary algorithms. The PVT collected time lapses, false starts, and reaction times.

**FIGURE 1:**
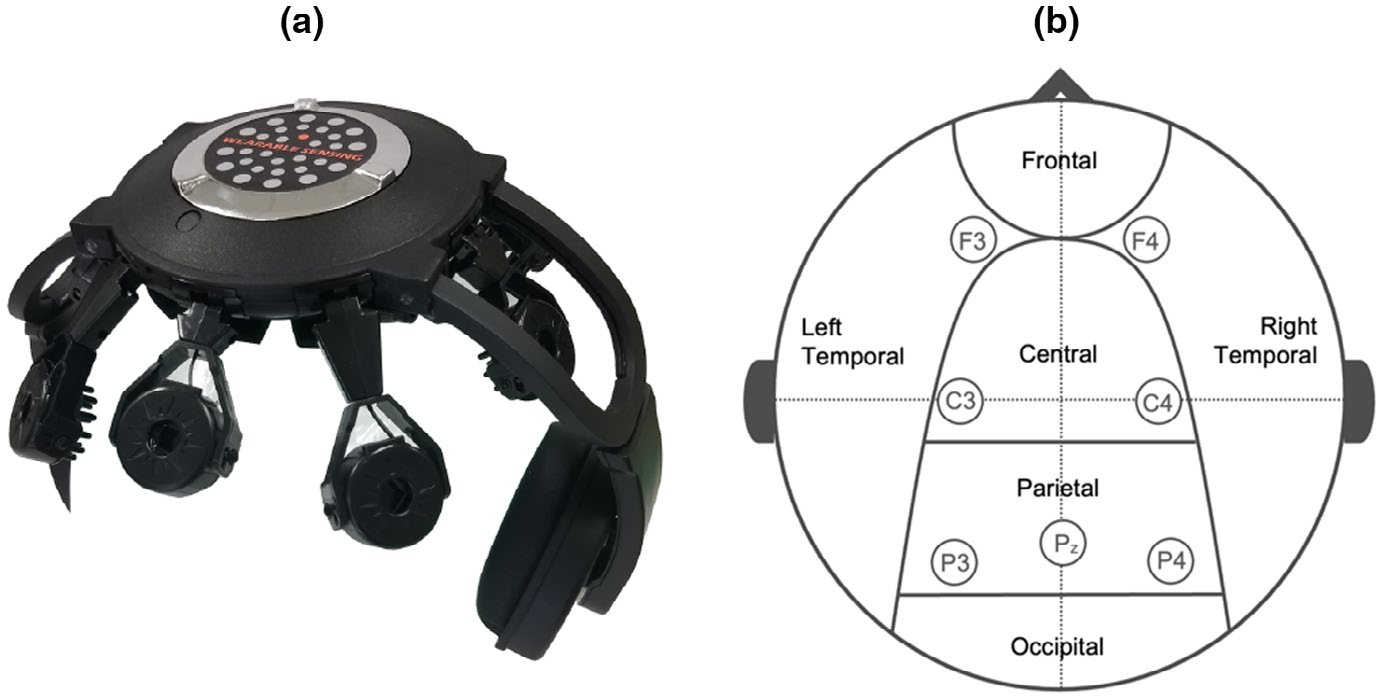
Displays the: (a) dry‐electrode EEG system (DSI‐7, Wearable Sensing, LLC, San Diego CA) and (b) the electrode placement used, with electrodes (F3, F4, C3, C4, Pz, P3, and P4) based upon the International 10–20 system.

The EEG device (see Figure 1, panel a) was fitted to the seated participant in accordance with the manufacturer's instructions. The acquisitions of EEG data were collected at a sampling rate of 300 Hz with the electrode setup (F3, F4, C3, C4, Pz, P3, and P4) abiding by the 10–20 International System (as displayed in Figure 1, panel b), and were pre‐filtered by the DSI‐7 system (DSI‐7, Wearable Sensing, LLC, San Diego CA). Pre‐filtering entailed the pass of a high‐pass filter of 1 Hz and a low‐pass filter of 50 Hz. The EEG reference electrode was placed upon electrode site LE. Once collected, all data were pre‐processed and analyzed using MATLAB_R2021a (TheMathWorks, Inc., Natick, Massachusetts, USA.; RRID:SCR_001622) and the EEGLAB 2021.1 package (Delorme & Makeig, 2004). Topographical maps were achieved through the statistical topographical neuro‐visualizing software available in EEGLAB (MATLAB Toolbox, California, USA; RRID:SCR_007292), represented by the default color map used by EEGLAB.

Sleep was monitored objectively using a Readiband (Fatigue Science Inc., Canada) accelerometer device worn on the nondominant wrist of each participant continuously over the whole study period (14 days). Algorithmically derived daily sleep duration data from the Readiband were cross‐compared each day with self‐reported bedtime and wake‐up data, and where discrepancies occurred, the self‐reported data were used.

### 2.5 EEG data processing

Each data collection underwent a uniform preprocess procedure, which involved data segmentation and the consecutive processing of Independent Component Analysis (ICA), whereby unwanted components were identified and removed. This was achieved both by filters and visual inspection‐based artifact rejection analysis (Delorme & Makeig, 2004), whereby any components that were predicted to account for non‐brain activity at a magnitude of equal to or above 90% were removed.

The Power Spectrum Densities (PSD) were quantified using Welch's Method, with windows of 500 sampling points and overlaps of 375 sampling points per dataset. The PSDs were extracted per participant per protocol and were decomposed into the following frequency bands: delta (1–3 Hz), theta (4–7 Hz), alpha (8–12 Hz), low beta (13–16 Hz), high beta (17–30 Hz), low gamma (31–45 Hz), and high gamma (55‐70 Hz) bands. PSD values were then log‐transformed for each dataset to achieve higher degrees of normal distribution; therefore, interpretations of individual frequency bands should be considered on respective scales. Relative Task Related Power values were used to baseline correct power values (Gyurkovics et al., 2021), which were achieved by subtracting the 1‐min resting protocol log‐transformed powers (P_i ref_) from the log‐transformed activation interval (P_i task_), using the following formula:

$\text{Task related power}\;\left(\mathrm{Log}\;P_{i}\right)=\log\;\left[P_{i\;\text{task}}\right]–\log\;\left[P_{i\;\mathrm{ref}}\right]$ (Pfurtscheller & Lopes da Silva, 1999).

The ORP was calculated by comparing the spectral analysis of EEG data segmented in 3‐s epochs, whereby the relative relationship between four EEG‐based frequency bands (delta, theta, alpha, and beta) was used, following the exact methodology described in detail elsewhere (Younes et al., 2015). Briefly, the 3‐s epochs are extracted from electrodes C3 and C4, and averaged, to deduce relative states of arousal within each participant. The association within each epoch, between each epoch, and between epochs of similar sleep scores is then assessed and used to provide an ORP score. This is an automated process using proprietary algorithms. Obtaining a lower ORP measure indicates a high association with the probability of deep sleep‐like states, whereas higher ORPs are associated with states of high arousal or waking states. An ORP of 1.25 involves an equal probability of being in either a sleep or waking state. ORP values are broken into the following stages of waking/sleep: Very Deep Sleep (≤0.25); Deep Sleep (>0.25, <0.50); Average Sleep (>0.50, <0.75); Light Sleep (>0.75, <1.00); Sleep Transition (>1.00, <1.75); Drowsy Awake (>1.75, <2.25); Fully Awake (≥2.25). Values were reported as the percentage (%) of time spent in each stage, per task.

For the investigation of the present study, the ORP values were further segmented into three primary stages: Asleep (≤1.0); Intermediate (Drowsy/Transition; >1.oo, <2.25); and Fully Awake (≥2.25). The outlined stages were adopted since we are not investigating sleep per se, rather, we are applying the ORP to *waking* EEG data; therefore, characterized stages of sleep were not of relevance for the current investigation. The % of time spent in each of these three stages was evaluated per task (1‐min eyes open resting, two oculography tests, 10‐min PVT, and 2‐min eyes‐closed procedure). Values were averaged across all participants, per each day (1–10).

### 2.6 Data and statistical analysis

Statistical analysis was conducted using SPSS version 29.0.0.0 (241; SPSS, Inc., Armonk, NY, USA; RRID:SCR_002865). Data were explored for all variables by calculating individual mean + 1 standard deviation as an individual threshold and using the 75th quartile from baseline week as a group threshold. Group statistics are shown as means ± standard deviation, unless stated otherwise. The alpha level for statistical significance was set at *α* ≤ 0.05. Confidence intervals (CIs) are presented at the 95% level to indicate the precision of estimates. The assumption of normality was evaluated using the Shapiro–Wilk test, and variables meeting the criteria proceeded to parametric testing. Cohen's *d* values were interpreted based on thresholds for small, moderate, and large effects (0.2, 0.5, and 0.8, respectively) (Cohen, 1988). Where appropriate, paired and independent samples *t*‐tests were selected over ANOVA due to the study's within‐subject design with two primary conditions (baseline vs. “live‐in”), allowing for a direct statistically robust comparison of mean differences while accounting for individual variability and maximizing power in a small sample (Mishra et al., 2019).

#### 2.6.1 Sleep

A paired samples *t*‐test was used to compare the baseline week with “live‐in” week, for the participants' average sleep duration (hours) per night, as obtained from discrete (i.e., self‐reported time in bed) and continuous (i.e., Readiband) scores.

#### 2.6.2 Cognitive tasks

Paired‐samples *t*‐tests were performed to compare the baseline week to the “live‐in” week, for each cognitive test: PVT and EyeSync variables, for all variables. PVT variables included lapses, median reaction times and false starts, and Oculography EyeSync variables involved using variance in radial, tangential, and horizontal metrics.

#### 2.6.3 EEG

Log transformed relative PSD (Task Related Power) values were used for the statistical comparisons for each frequency band for each cognitive task, and log transformed PSD (μV^2^/Hz) values were used for the statistical comparisons of the waking‐rest eyes‐closed condition.

For the cognitive tasks, a paired samples *t*‐test was conducted for each EEG frequency band to compare mean Task‐Related Power values between the “live‐in” and baseline weeks during both the PVT and EyeSync protocols. Paired and Independent samples *t*‐tests were conducted to investigate the main statistical effects of sleep across each PSD band within the waking‐rest 2‐min eye‐closed condition. The paired samples *t*‐tests compared the mean difference (MD) between (a) week 1 and week 2 PSD values, (b) the week 1 and week 2 am PSD values, and (c) week 1 and week 2 pm PSD values, and the independent samples *t*‐tests compared the MD in theta PSD values for (d) all am and pm across both weeks, (e) week 1 am and pm sessions, and (f) week 2 am and pm session.

EEG data sets were also evaluated through statistical topographical maps using EEGLAB parametric statistics, which projected statistical planar positions of the hemispheric scalp model, evaluating the theta band statistics of all participants across the baseline (week 1) and the ‘live‐in’ (week 2) weeks, for the eyes‐closed and the PVT protocol.

#### 2.6.4 Odds ratio product

Independent samples *t*‐tests were conducted to compare the % of time spent in the denoted three ORP stages. The initial evaluation compared the baseline versus live‐in week for all tasks (i.e., 1‐min eyes open resting, two oculography tests, 10‐min PVT, and 2‐min eyes‐closed procedure). Such evaluation was performed to determine if the detection of accumulated fatigue between the weeks was found in waking performance, as per the ORP. A follow‐up evaluation compared the first to last days of both weeks (i.e., days 1 vs. 5, and days 6 vs. 10) and each week independently for all tasks (i.e., 1‐min eyes open resting, two oculography tests, 10‐min PVT, and 2‐min eyes‐closed procedure). This evaluation was performed to determine the sensitivity of detection of accumulated fatigue throughout a week, as per the ORP.

Direct differences between corresponding days of the week (i.e., first days of the week; day 1 vs. day 6, second days of the week; day 2 vs. day 7, etc.) were evaluated using paired samples *t*‐tests across all tasks within each ORP stage. Such evaluation was performed as a direct individual comparison between days of the week, from the baseline to the live‐in week.

## 3 RESULTS

### 3.1 Sleep

Participants' average sleep duration (hours) per night was significantly greater for the baseline week (7.4 ± 0.7 h) compared to “live‐in” week (5.4 ± 0.1 h) (*p* < 0.01). Sleep efficacy was not detected to be significantly different between the baseline week (86.2 ± 12.3%) to the “live‐in” week (89.1 ± 9.8%).

### 3.2 Psychomotor vigilance task

**FIGURE 2:**
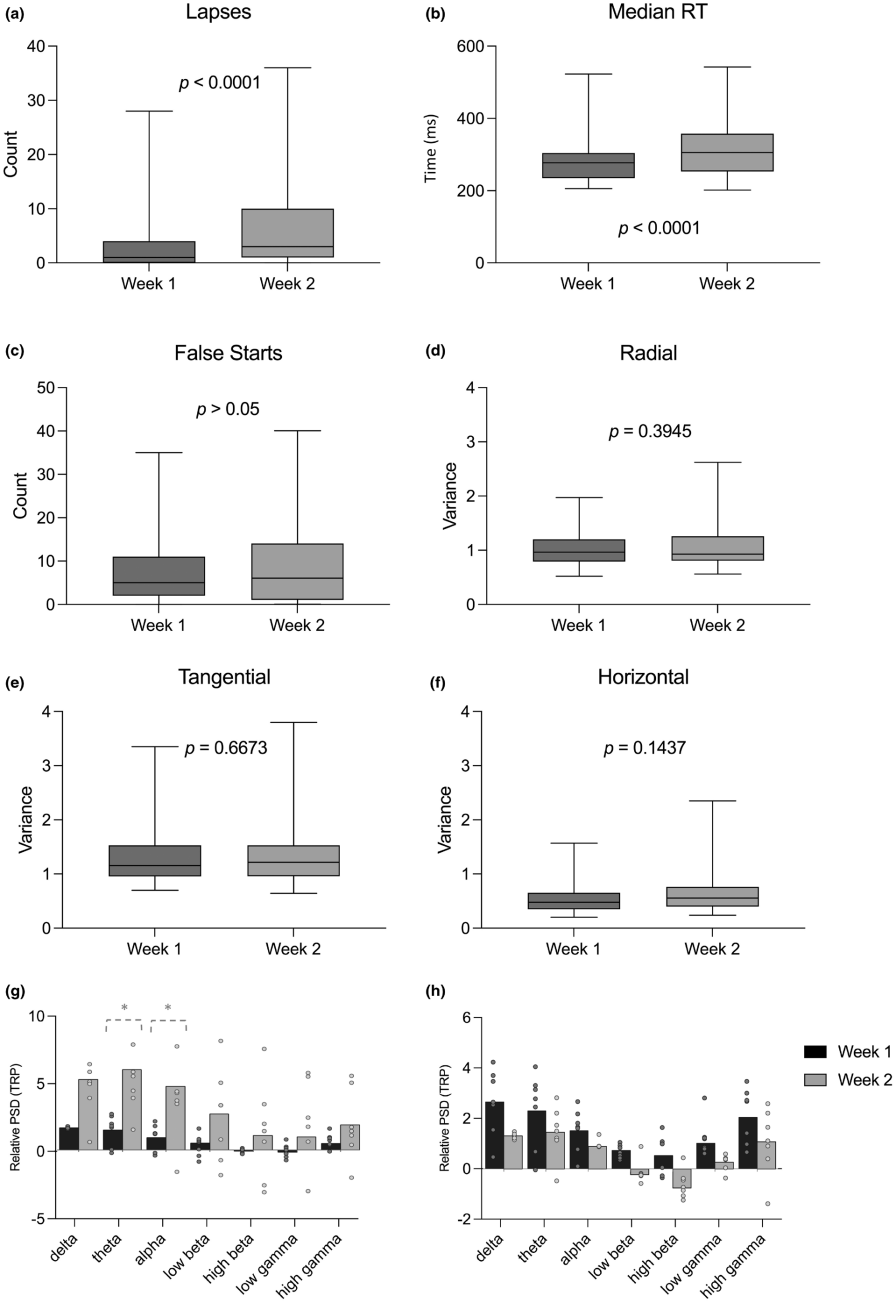
Difference between baseline (week 1) and “live‐in” (week 2) are displayed for the; 10‐min Psychomotor Vigilance Task (PVT‐10), comparing averaged lapses (panel a), median reaction times (RT) (panel b), and false starts (panel c); Oculography data from the Eye‐Sync device for the radial (panel d), tangential (panel e), and horizontal (panel f) results. The normalized Relative Power Spectrum Density (PSD; Task Related Power [TRP]) values plotted against the frequency bands, comparing the baseline week (week 1) versus the “live‐in” week (week 2) for the Psychomotor Vigilance Task (PVT) protocol (panel g) and EyeSync protocol (panel h). Box and whisker plots show ranges from the minimum to maximum values for each dataset. The interquartile range (IQR) spans from the 25th to the 75th percentile, representing the spread of the middle 50% of the data. Whiskers indicate the overall data range. Error bars represent 1 standard deviation. * denotes *p* < 0.05.

Significant group‐level performance decline was observed when comparing lapses and median reaction times from baseline week (week 1) to “live‐in” (week 2). No statistical differences were found for the false starts. Difference between baseline (non‐fatigued; week 1) and “live‐in” (fatigued; week 2) for the 10‐min PVT are found for the lapses, median reaction times and false starts, in Figure 2 (panels a, b, and c, respectively).

### 3.3 Psychomotor vigilance task EEG

From the baseline to “live‐in” week, statistically significant increases were reported in the theta (*t* (29) = 2.308, *p* = 0.028, *d* = 0.421, CI = [0.528, 8.755]) and alpha (*t* (29) = 2.124, *p* = 0.042, *d* = 0.388, CI = [0.144, 7.852]) bands, for the Task Related Power values during PVT (Figure 2g).

### 3.4 EyeSync

Within the group‐level performance, no significant differences were noted when comparing oculography performance from baseline week (week 1) to “live‐in” (week 2). Comparisons between baseline (non‐fatigued; week 1) and “live‐in” (fatigued; week 2) for oculography performance are found in Figure 2 (panels d, e, and f).

### 3.5 EyeSync EEG

Mean frequency band Task Related Power values are shown in Figure 2h; there were no statistical differences (*p >* 0.05) found in any bands.

### 3.6 Eyes‐closed EEG

**FIGURE 3:**
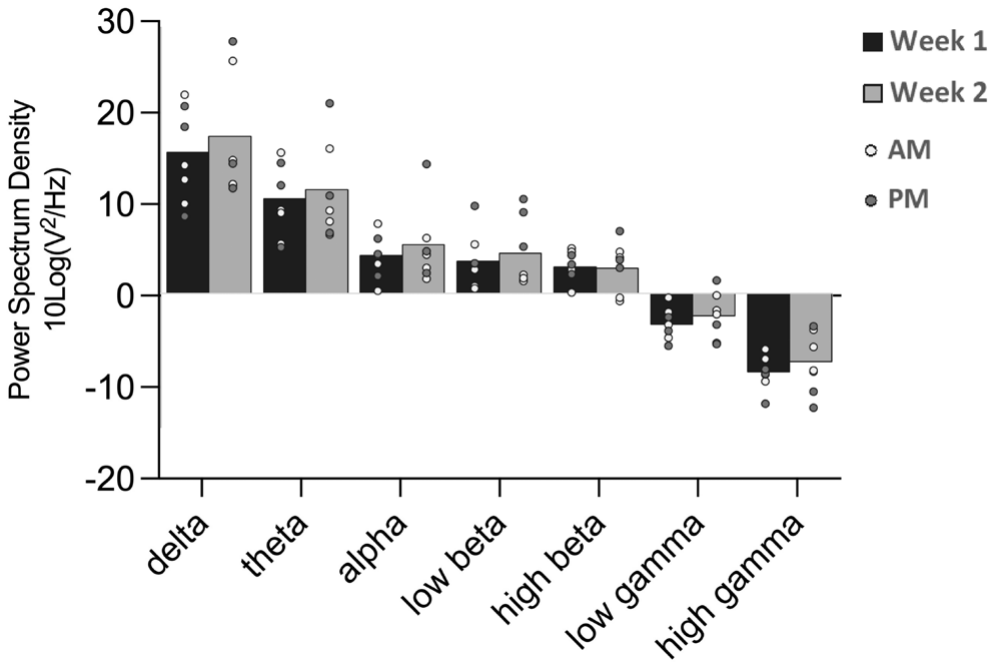
Average log transformed Power Spectrum Density (PSD) (μV^2^/Hz) values plotted against all frequency bands, comparing the baseline week (week 1) to the “live‐in” week (week 2) for the eyes‐closed protocol, relative to baseline. Within the graph, averages of each participant are presented per week, and are denoted as am or pm accordingly.

Week (baseline vs. “live‐in”) and the time of day (am vs. pm) for the waking‐rest 2‐min eyes‐closed PSD activity were compared. No statistically significant effects were found in PSD values across all frequency bands when comparing the mean PSD differences from the baseline to the “live‐in” week (Figure 3). Additionally, no significant changes in PSD values were observed between the am sessions of week 1 and week 2, nor between the baseline and “live‐in” periods during the pm sessions. No other significant changes were noted.

### 3.7 Odds ratio product

**FIGURE 4:**
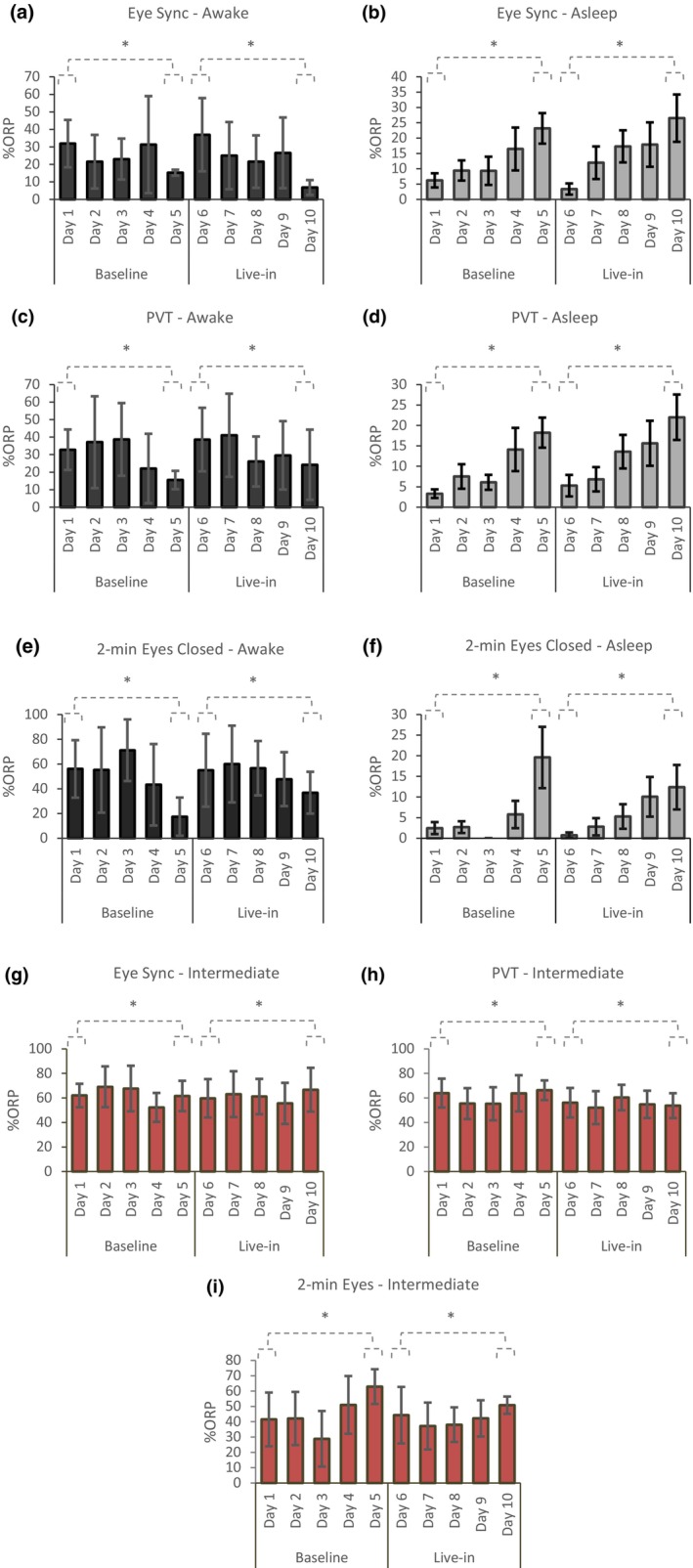
Difference between baseline (week 1) and “live‐in” (week 2) are displayed for the; Oculography task (Eye‐Sync), displaying the percentage (%) of total task time spent fully awake (panel a) and asleep (panel b); the 10‐min Psychomotor Vigilance Task (PVT‐10), displaying the percentage (%) of total task time spent fully awake (panel c) and asleep (panel d); the 2‐min eyes closed protocol displaying the percentage (%) of total task time spent fully awake (panel e) and asleep (panel f). The percentage (%) of total task time spent in an intermediate (drowsy/transition) stage is displayed for the Oculography task (Eye‐Sync), PVT‐10, and 2‐min eyes closed in panels g, h, and i, respectively. * denotes *p* < 0.05.

The ORP evaluation revealed no significant difference between weeks across all waking tasks (*p* > 0.05). This was neither found for the % of time spent asleep, fully awake, nor the % of time within an intermediate stage. Follow‐up investigation revealed that when comparing the ORP values of the first to the last days of the weeks, it was deduced that ORP was sensitive enough to detect the changes in % of time spent in each stage within a week, with the amount of time spent fully awake decreasing (MD = 27.5%, SD = 13.23%, *t* (75) = 5.037, *p* < 0.001, *d* = 1.33 CI = [17.466, 37.534]), across all tasks (Figure 4a,c,e). Particularly, a 28.44 ± 12.00% decrease was found in the baseline week for the % of time spent fully awake from the first to the last day (i.e., day 1 to day 5), whereas a 23.29 ± 19.72% decrease was found in the live‐in week (i.e., day 6 to day 10).

The inverse was found for the % of time spent in a sleep state within each week across waking tasks (day 1 vs. 5 and day 6 vs. 10; MD = 14.3%, SD = 6.61%, *t* (71) = 5.820, *p* < 0.001, *d* = 1.44, CI = [9.418, 19.235]), whereby progressive increases in % of time asleep was found in all tasks (Figure 4b,d,f). A 17.14 ± 4.25% increase was found in the baseline week for the % of time spent characterized in a sleep state from the first to the last day, with a 12.97 ± 3.89% increase in the live‐in week. No distinct patterns were displayed for % of time spent in the characterized intermediate stage, neither between weeks nor within tasks (Figure 4g,h,i). Although, an increase in the % of time spent in the characterized intermediate stage increased when comparing the first to the last days of the week across all tasks (MD = 10.0%, SD = 4.6%, *t* (75) = 2.174, *p* = 0.033, *d* = 0.53, CI = [0.837, 19.222]).

Percentage changes of ORP stages between corresponding days of the weeks revealed no detected significant differences (*p* > 0.05), apart from the % of time spent in an asleep stage on the fourth day of the weeks (i.e., day 4 vs. day 9; MD = −3.56, SD = 9.11, *t* (27) = −2.07, *p* = 0.049, *d* = −0.39, CI [−0.770, −0.002]). Mean differences ranged from 9.29% to 2.05%, with the largest change in being observed in % of time characterized as being fully awake on the last days of the week (i.e., day 5 vs. day 10; MD = −9.29, SD = 18.97, *t* (11) = −1.70, *p* = 0.118, *d* = −0.49, CI [−1.080, 0.120]). Overall, these results suggest minimal significant differences between paired conditions, with small effect sizes and limited evidence of systematic changes as characterized by the ORP.

### 3.8 Topographical maps

**FIGURE 5:**
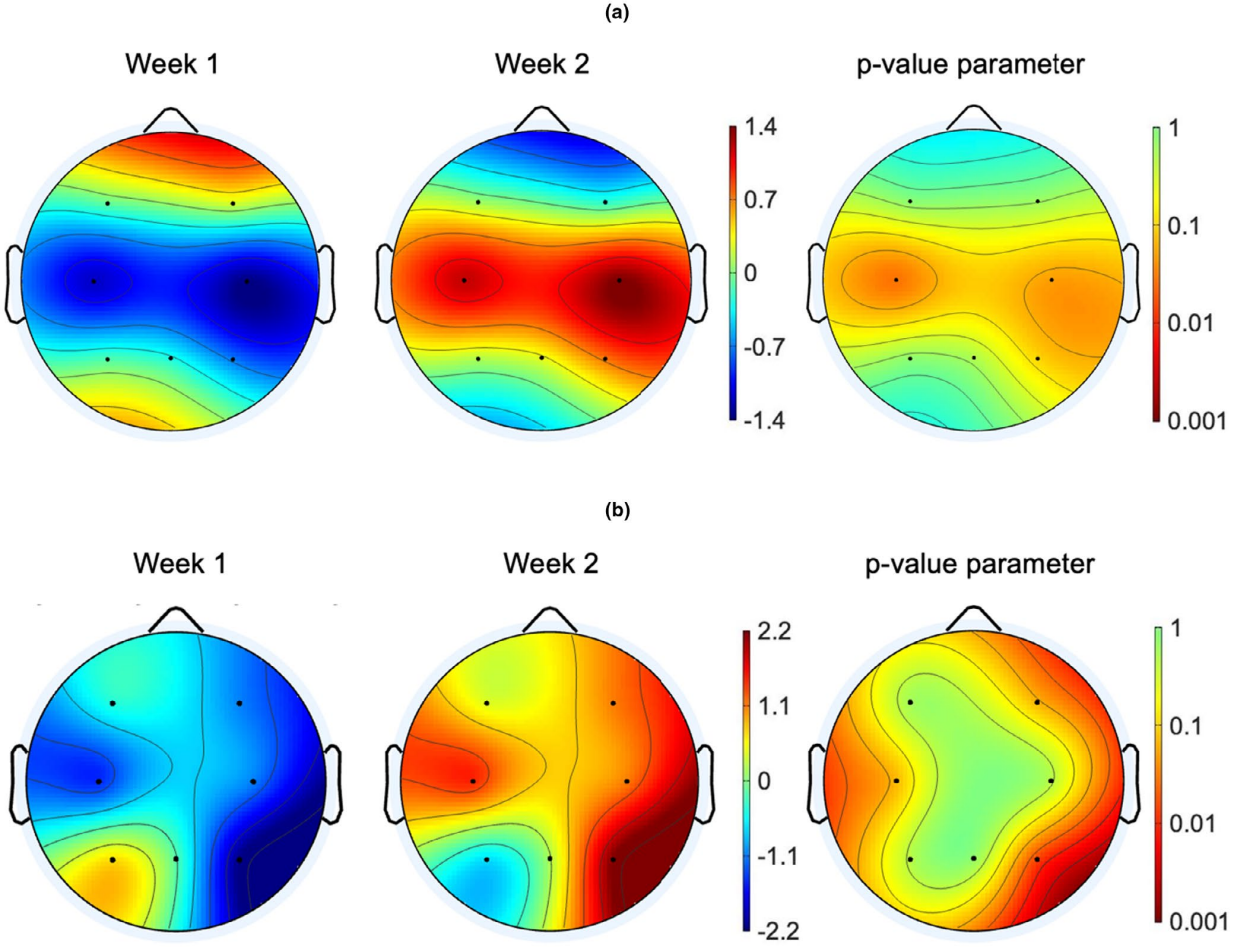
The normalized parametric statistic topographical comparison of the baseline week (week 1) and the “live‐in” week (week 2) for the (a) eyes‐closed protocol and (b) Psychomotor Vigilance Task (PVT) protocol. These topographical plots display the normalized Power Spectrum Densities (μV^2^/Hz) within the theta band (4–7 Hz) for week 1, week 2, and the *p* value parameter, respectively.

Normalized parametric statistics topographical maps were used to evaluate statistical contributions of electrode sites, comparing the baseline week (week 1) and the “live‐in” week (week 2) for both the eyes‐closed and PVT protocol within the theta band (4–7 Hz). As displayed in Figure 5, no statistical difference (*p >* 0.05) was found on either electrode sites across the 2 weeks for the eyes‐closed condition within the theta band, although greatest difference was found within the mid region of the brain on electrodes C3 and C4. Within the PVT protocol, statistical difference was found in the right parietal lobe on electrode site P4 within the theta band, indicating that this electrode had the greatest contribution in the identified increase in PSD in the PVT protocol within the theta band, when comparing the “live‐in” to the baseline week.

## 4 DISCUSSION

The primary aim of this study was to evaluate the neural correspondences associated with a PVT test and EyeSync oculography tests, as well as to evaluate the neuronal activity in waking‐rest (i.e., eyes‐closed) states with an increase in sleep propensity due to increased workload and overall decreased sleep duration. As a result, this study aimed to verify the EEG theta band as a marker of waking sleep‐deprived states, as well as to provide insight on the neurological correspondences involved in wake‐dependent cognitive performance. The findings confirmed that cumulative sleep loss (5.4 ± 0.1 vs. 7.4 ± 0.7 h) and increased workload were associated with significant increases in theta (*p* = 0.028) and alpha (*p* = 0.042) power spectrum densities during a cognitive task, along with declining Odds Ratio Product (ORP) values, highlighting these markers as potential indicators of fatigue in operational contexts.

### 4.1 Low frequency band investigation during cognitive tasks

It was hypothesized that within both cognitive tasks, lower frequency waves (i.e., delta and theta waves) would most prominently be impacted by the protocol, compared to higher frequency waves. The results of the present study are in partial support of this, with the theta and alpha bands in the PVT protocol showing a significant increase in activity compared to the baseline week. Although all other frequency bands were found to increase within the PVT protocol, the theta and alpha frequency bands were the only which were statistically different.

Previously, both positive and negative results have been found when evaluating the relationship between neuronal activity and diminishing behavioral performance due to states of SD. For instance, an increase in absolute (4–20 Hz) spectral power, particularly in the left central cortex, has been associated with decreased PVT performance (Corsi‐Cabrera et al., 1996), although a more recent study found no correlation between EEG and PVT performance as a response to SD‐based protocols (Caldwell et al., 2003). Overall, previous findings suggest a positive correlation between the lower frequency band (i.e., delta and theta) power densities and PVT performance (Cajochen et al., 1999; Gorgoni et al., 2014; Hoedlmoser et al., 2011), and have resulted in the theta band being proposed as a salient marker of declined PVT performance associated with sleep‐deprived states (Gorgoni et al., 2014). The results of the present study support this claim, with the theta band within the PVT study showing the greatest increase compared to the delta and alpha band. Interestingly, since all frequency band spectral values increased compared to prior conditions in states of SD apart from the high‐beta frequency band (16–24 Hz) (de Gennaro et al., 2007; Finelli et al., 2000; Gorgoni et al., 2014; Tinguely et al., 2006), the PVT results support this, as the least reported difference in the present study was found within the high beta band.

Furthermore, during PVT, a recent study investigating the impact of SD on the brain found the theta band to increase in all cortical regions, with particular emphasis on a positive correlation between PVT performance and subjective sleepiness with EEG theta activity in the centro‐posterior region (Gorgoni et al., 2014). Interestingly, Smith et al. (2002) has also noted that lower frequency bands (delta and theta) increase in posterior regions in resting awake conditions. The statistical topographical maps within the current study act to indicate that the right occipital region was likely of contribution to this activity as the present results displayed greatest electrode contribution on site P4. As this present study is only indicative of partial support of the literature, further evaluation is suggested with a larger sample size in order to substantiate such claim.

Within the EyeSync oculography protocol, all frequency bands were found to decrease in the “live‐in” week compared to the baseline week, although these decreases did not reach statistical significance. The inverse relationship found in the neural response of the EyeSync test compared to the PVT (i.e., decrease in activity rather than overall increase), is proposed to derive from the differences in demands of these tasks. For instance, the PVT task requires continuous attention, whereas the EyeSync is a repetitive task which can, to some degree, be learnt, and thus is proposed to require less neural resources in order to execute with time (Dunst et al., 2014). Potentially other EyeSync oculography tests involving random appearance of pixels may induce similar EEG responses to the PVT, reflecting a difference in neural demand compared to those presently tested. As no previous studies to our knowledge have successfully reported the neuronal correlates associated with the cognitive decline in performance measured using the EyeSync and EEG, further investigation is recommended to provide insight on the neural markers of SD using this particular method of ocular tracking.

### 4.2 Neuronal correspondences of reduced sleep duration on waking‐rest (eyes‐closed) conditions

It was also hypothesized that decreased sleep duration would associate with increased overall waking‐rest (eyes‐closed) theta activity. The results of the present study concur with this hypothesis, with the highest detected difference in activity in the mid region of the brain (electrodes sites C3 and C4), albeit not to a significant degree. Previous EEG studies have shown nearly all frequency bands to increase in post‐SD conditions, although the most prominent changes are reported to be in the 0.75–9 Hz and 11–16 Hz frequency ranges (de Gennaro et al., 2007; Gorgoni et al., 2014; Jones & Harrison, 2001; Lim & Dinges, 2008). With the present study finding the largest statistical contribution within electrode sites C3 and C4 in the eyes‐closed (waking‐rest) protocol, the present results are in alignment with the literature, which found the greatest activation exhibited within the frontal and central regions (de Gennaro et al., 2007; Finelli et al., 2000; Gorgoni et al., 2014; Lorenzo et al., 1995; Strijkstra et al., 2003; Tinguely et al., 2006). Although low frequency activity in frontal regions have been noted to be a prominent feature of SD EEG (Cajochen et al., 1999; Horne, 1993; Oken et al., 2006), Lorenzo et al. (1995) reported the central regions of the brain (C3) to show the greatest significant increase in theta activity within the eyes‐closed conditions, which the present findings are in support of.

### 4.3 am versus pm theta band activity

The third hypothesis involved theta activity being higher in the pm group in the “live‐in” week, for the direct relationship the theta band has been found to have with the time elapsed since waking. As this has been found both in states of waking‐rest (de Gennaro et al., 2007; Finelli et al., 2000; Tinguely et al., 2006) and during cognitive tasks such as the PVT (Gorgoni et al., 2014), the theta band has thereby been denoted as a marker for sleep propensity (de Gennaro et al., 2007; Finelli et al., 2000; Gorgoni et al., 2014; Tinguely et al., 2006). Although the overall pm session theta PSD values were higher than overall average am session values across both weeks, this was not found to a significant degree.

As these results are reflective of a small sample size, it is notable that this investigation would benefit from having all participants completing both am and pm sessions. Furthermore, although all cognitive tests were performed in both the am and pm sessions, EEG was only administered once per participant per day due to the time sensitivity associated with the Military Clearance Diver course schedule. As half of the participants had their EEG data collected during the am session and the other half during the pm, a greater sample size per am and pm session would have allowed for a higher representative comparison of sleep propensity on cognitive performance and associated neuronal measures and may have resulted in stronger support of the current literature.

### 4.4 Odds ratio product

The ORP is a validated continuous variable that employs EEG frequency band powers as a means of determining a single index of sleep and has been determined to be highly responsive and correspondent to detecting sleep‐like/arousal states (Younes et al., 2015). Although the predominant use of the ORP thus far has been targeted for sleep‐like states, the present study aimed to evaluate the tool's transferability toward waking. In doing so, the last hypothesis entailed a decline in waking ORP values being found with cumulative sleep loss.

Across all tasks (i.e., PVT, EyeSync, and 2‐min eyes closed), the % of total time spent “asleep” was found to increase, and the % time of total task spent “fully awake” decreased across both the baseline (day 1–5) and live‐in week (day 6–10). These results reveal that the ORP offers the potential for detecting increases in cumulative fatigue throughout a week. As this pattern was revealed in the EEG data collection of 2‐min eyes closed, this indicates that a cognitive task may not be required to detect cumulative fatigue displayed within a week. Therefore, such results indicate that a short 2‐min interval of eyes closed EEG may be sufficient in supporting the detection of cumulative fatigue endured throughout a week.

Since no significant difference between ORP values was found across weeks, these results could be attributed to the calculation of the ORP, which has been developed and validated using EEG data derived during sleep. Therefore, further validation of the tool within waking EEG is required, for example by applying the ORP algorithm to a range of data collected during waking states. Additionally, it is important to note that as weekend workload and sleep patterns were not accounted for within the present study (i.e., between days 6 and 7), this could account for a “resetting” of baseline ORP % value. If the weekend workload and sleep patterns were controlled for (i.e., continuing the sleep restriction over the weekend), it is assumed that a continuous decrease in % of time spent awake per task, and an increase of % of time per task spent asleep, would be found. Overall, promising trends were identified in the detected total time spent “asleep” per task, across both cognitive tasks (EyeSync and PVT‐10) and the 2‐min eyes closed protocol. Importantly, the ORP data suggest a within‐week pattern in sleepiness regardless of nocturnal sleep achieved, which highlights the potential importance of avoiding an accumulation of workdays without a day off to maintain awareness during critical cognitive tasks. Although further evidence to support the ORP's application within waking states is required. The specific duration of time off‐task required to restore optimal ORP data on‐task is a subject for future investigation.

### 4.5 Strengths and limitations

To our knowledge, the current study is one of the first to employ a dry‐electrode EEG system for the purpose of detecting waking cognitive performance‐based neuronal markers associated with states of increased workload and reduced sleep duration. The DSI‐7 is a novel dry‐electrode system that requires no gels or fluids on the scalp to collect data, which offers promise for the implementation within clinical and operational settings (Lopez‐Gordo et al., 2014). In addition, with recruiting a specialist population of Military Clearance Diver trainees, the present study thereby offers valuable insight into the detection of modulation in waking neuronal activity, which may act to support characterizable fatigue‐based markers for the implementation within operational and clinical settings.

Although current dry‐electrode systems offer great potential practical application for the detection of SD‐based markers, it must be noted that present devices currently remain somewhat cumbersome, as well as require substantial data processing is required to convert raw data into user‐friendly information. Additionally, the limited number of electrodes restricts the informativeness of the topographical maps. It should also be noted that within the present study, weekend sleeping patterns were not controlled for, nor was data collected during these periods. As these sleeping patterns are likely to vary per participant in alignment with their plans and schedules, consecutively controlled and monitored sleep regimes is suggested to provide more insight on the cumulative impact of SD in such protocol. In addition, this investigation would have also benefited from a larger sample size. Lastly, the current study focused on identifying neural markers associated with the cognitive tasks, rather than examining direct correlations between EEG activity and performance metrics, due to the modest sample size and the complexity of task‐specific neural dynamics. Future research with larger samples is needed to explore these relationships more rigorously. With this stated, it is argued that the results provide valuable insight on the potential neuronal alterations endured as a result of states of reduced sleep duration, and that further investigation on the basis of these results is warranted.

### 4.6 Practical applications

The EEG is a practical and highly accessible monitoring technique that provides a dynamic assessment of the brain‐based activity (Tatum & Ellen, 2014). Although traditional wet electrode EEG systems are commonly employed to quantitatively measure brain activity and offer greater practical use compared to alternative measuring techniques (i.e., Functional Magnetic Resonance Imaging [fMRI] and Polysomnography [PSG] (Tatum & Ellen, 2014)), several limitations make it challenging to apply these within operational settings. For instance, traditional EEG systems have been deemed impractical due to the time and inconvenience of the wet electrode systems compared to alternative advancements in EEG systems, such as dry‐electrode EEG systems (i.e., within clinical settings (Hinrichs et al., 2020)).

The recent development of dry‐electrode technology has dramatically reduced the time required to set up data collection and therefore potentially removed this barrier and reignited interest in this field, all while remaining the integrity of detectable signal (Hinrichs et al., 2020; Leach et al., 2020). The technological advancement of dry‐electrode EEG allows for application times of only a few minutes, offering an ease in use, and results in less mess. This therefore supports the facilitation of rapid EEG data collection, creating new opportunities for operational setting‐based implementation (Drollinger et al., 2019; Rice et al., 2019). In addition, dry‐electrode EEG systems have even shown potential for the real‐time detection of drowsiness, which was used to alert drivers falling under a threshold of alertness (Tsai et al., 2009).

Consequently, as noted above, present dry‐electrode EEG systems currently require advancements in design application in order to support integration and practical use, as well as require more user‐friendly approaches of data analysis to support the implementation of data within the scope of those using the equipment, that is, real‐time decision making. With this stated, although early in research, the dry‐electrode system could be used as a method for detecting diurnal sleep‐related fatigue, noting that refinements in optimal approach with further applied studies are needed.

## 5 CONCLUSION

The present study investigated the neuronal correlates of decreased sleep duration on waking states at rest and during two cognitive performance tasks in Military Clearance Diver trainees, demonstrating detectable modulation in waking neuronal activity in response to a decrease in sleep and an increase in workload. Specifically, significant increases in theta (*p* = 0.028) and alpha (*p* = 0.042) power spectrum densities during a cognitive task were observed during periods of reduced sleep and increased workload, alongside declining within‐week Odds Ratio Product (ORP) values, highlighting these metrics as potential indicators of fatigue. These findings provide a foundation for characterizing fatigue‐based markers for implementation within operational settings.

By employing a novel dry‐electrode EEG, the study demonstrated the device's potential as a quick and easy‐to‐administer tool for neuronal detection, offering promise for identifying waking neural correlates associated with reduced sleep duration. Combining this technology with a sleep/wake index scale (such as the ORP) offers practical potential for detecting fatigue‐based markers in operational contexts, particularly in military settings. While the dry‐electrode EEG system offers numerous advantages over traditional wet‐electrode approaches, further applied studies are needed to refine and optimize this approach.

## AUTHOR CONTRIBUTIONS

J. Morrone was involved in conceptualization, EEG analysis, methodology, writing‐original draft, writing‐review and editing. S. Coakley and C. Pedlar were involved in conceptualization, design, data collection, writing‐review and editing. S. Blacker, S. Myers, N. Stanley, and S. Halson were involved in conceptualization, design, writing‐review and editing. P. Hough, C. Vine, and T. Maroni were involved in data collection, writing‐review and editing. A. Siddall, S. Patterson, M. Jones, and K. Chillingsworth were involved in writing‐review and editing.

## FUNDING INFORMATION

This research project was funded by the Ministry of Defence (UK) and managed by the Defence Science Technology Laboratory (Dstl); it was contracted through the Human Social Science Research Capability (HSSRC).

## CONFLICT OF INTEREST STATEMENT

All authors have no conflicts of interest.

## ETHICS STATEMENT

Ethics approval was provided by the Ministry of Defence Research Ethics Committee (MoDREC, Protocol Number 2088/MODREC/21).

## Data Availability

The data that support the findings of this study are available via the corresponding author upon request.
